# Determination of lower cut‐off levels of adalimumab associated with biochemical remission in Crohn's disease

**DOI:** 10.1002/jgh3.12266

**Published:** 2019-10-06

**Authors:** Arne Carlsen, Roald Omdal, Lars Karlsen, Jan Terje Kvaløy, Lars Aabakken, Øyvind Steinsbø, Nils Bolstad, David Warren, Knut Erik Aslaksen Lundin, Tore Grimstad

**Affiliations:** ^1^ Gastroenterology Unit, Department of Internal Medicine Stavanger University Hospital Stavanger Norway; ^2^ Department of Clinical Science, Faculty of Medicine University of Bergen Bergen Norway; ^3^ Clinical Immunology Unit, Department of Internal Medicine Stavanger University Hospital Stavanger Norway; ^4^ Department of Mathematics and Physics University of Stavanger Stavanger Norway; ^5^ Research Department Stavanger University Hospital Stavanger Norway; ^6^ Department of Transplantation Medicine Oslo University Hospital Rikshospitalet Oslo Norway; ^7^ Department of Medical Biochemistry Oslo University Hospital Radiumhospitalet Oslo Norway; ^8^ KG Jebsen Celiac Disease Research Centre University of Oslo Oslo, Norway

**Keywords:** adalimumab, Crohn's disease, lower cut‐off, remission, therapeutic drug monitoring

## Abstract

**Background and Aim:**

Adalimumab is administered and dosed using a standardized treatment regimen. Although therapeutic drug monitoring (TDM) may help optimize treatment efficacy, the lower cut‐off concentration of adalimumab needed to retain disease remission has not been established. This cross‐sectional study of patients with Crohn's disease on stable medication aimed to determine a lower therapeutic drug concentration threshold of adalimumab associated with biochemical disease remission.

**Methods:**

C‐reactive protein (CRP) and fecal calprotectin were used as established markers and albumin as an explorative marker of disease activity. Time since introduction, treatment interval, drug dosage, serum drug concentration and antidrug antibodies, disease duration, age, and sex were recorded.

**Results:**

The study included 101 patients who were divided into “active disease” and “remission” groups for inflammatory markers based on cut‐off levels of 5 mg/L for CRP and 50 mg/kg for fecal calprotectin. Cut‐off levels for albumin of 36.5 and 41.5 g/L were also added as further indicatives of remission. Receiver operating characteristic analysis found optimal thresholds for adalimumab associated with remission at 6.8–7.0 mg/L for the combination of CRP and fecal calprotectin and when combining CRP, fecal calprotectin, and albumin.

**Conclusions:**

In patients with Crohn's disease, serum adalimumab of at least 6.8 mg/L was associated with biochemical disease remission based on CRP and fecal calprotectin, supporting the use of TDM to ensure disease control. Albumin should be further tested in this setting.

## Graphical abstract

In this article, we examined the lower adalimumab serum concentration levels found to be associated with biochemical remission in patients with Crohn's disease. Serum adalimumab level of at least 6.8 mg/L was associated with biochemical disease remission based on C‐reactive protein (CRP) and fecal calprotectin, supporting the use of therapeutic drug monitoring (TDM) to ensure disease control.

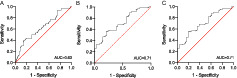

## Introduction

Crohn's disease is a chronic inflammatory disease of the gastrointestinal tract that is characterized by transmural and discontinuous inflammation of the intestinal wall. Common complications of Crohn's disease include strictures, fistulas, and abscesses, and surgery is frequently required to address these complications.^1^, ^2^ Over the last few decades, biological agents have emerged as a pivotal treatment option in moderate to severe inflammatory bowel disease.^3^ The fully humanized monoclonal antitumor necrosis factor (TNF)‐α agent adalimumab (ADL), which is administered subcutaneously using a standardized treatment regimen, is well documented to induce and maintain disease remission in moderate to severe Crohn's disease.^4^, ^5^, ^6^

Lack of response and loss of response represent major challenges in anti‐TNF‐α treatment. Primary nonresponse, defined as insufficient clinical response after induction therapy, is reported in about one‐third of the patients. Furthermore, even after an initial treatment response, as many as half of all patients experience a secondary loss of response at some point during the maintenance phase.^7^ The concept of therapeutic drug monitoring (TDM), which entails measuring serum drug concentrations and antidrug antibodies (ADAs) in order to guide treatment, allows an individualized approach to the dosing regimens of biologic agents. TDM can provide the information needed to optimize treatment and a better rationale for changing the treatment strategy in patients with an inadequate response or a loss of response. TDM thus has the potential to improve both drug efficacy and drug safety.^8^, ^9^, ^10^, ^11^

Even though TDM is increasingly available to clinicians in everyday use, evidence regarding the therapeutic consequence of TDM remains sparse and conflicting.^12^, ^13^, ^14^, ^15^, ^16^ An association between serum ADL concentrations and disease activity has been reported, using lower therapeutic cut‐off levels (in the range of 5–8 mg/L) to maintain treatment response/remission.^12^ However, the optimal ADL serum concentration ranges in Crohn's disease are not yet firmly established.

In this study, we used C‐reactive protein (CRP) and fecal (f‐) calprotectin (established markers) and serum albumin (explorative marker) as distinct markers of inflammatory activity in Crohn's disease patients on maintenance treatment. We aimed to identify a lower cut‐off serum level associated with biochemical remission in ADL‐treated patients.

## Patients and methods

### *Patients and study design*

This cross‐sectional study, which was conducted at Stavanger University Hospital, Stavanger, Norway, included patients who were diagnosed with Crohn's disease and were at least 16 years old. The patients were recruited from the outpatient clinic at the hospital. Included patients were receiving maintenance treatment with ADL, with a minimum of three doses administered before inclusion. The patients had one study visit along with their regularly scheduled trimonthly medical checkup from 1 April to 30 September 2014. The exclusion criteria were inability to provide consent and inability to adhere to the study protocol. The patients described here belong to a larger IBD cohort, and some descriptive data have been reported previously.^17^

### *Inflammatory markers*

CRP and albumin concentrations in blood were measured at the study visit and analyzed using the CRP VARIO 6K2641 and Serum Albumin BCP (Bromocresol purple), Architect c16000TM (Abbott Diagnostics, Lake Forest, IL, USA); f‐calprotectin was extracted from first morning defecation within 3 days before and 3 days after the study visit and was quantified using the automated EliA Calprotectin 2 enzyme fluoroimmunoassay (Phadia/Thermo‐Fischer, Uppsala, Sweden).

Remission *versus* active disease was categorized biochemically by CRP > 5 mg/L and/or f‐calprotectin > 50 mg/kg. Patients were consequently divided into an *active disease* group and a *remission* group for each marker. These cut‐off levels were applied for analysis of ADL levels and for developing the receiver operating characteristic (ROC) models. To optimize the ROC model, we also used a combination of CRP and f‐calprotectin as a composite disease activity marker.

We also explored albumin as a surrogate marker for remission *versus* active disease. Based on albumin quartile analysis, we selected two different cut‐off values representing the limits toward the lowest (36.5 g/L) and the highest (41.5 g/L) albumin quartiles. We assumed that the lower albumin quartile levels included the patients with the most severe inflammation, while the upper albumin quartile levels represented the patients with the least inflammatory burden. These albumin cut‐offs were used to develop two different explorative composite/combined ROC models, including CRP, f‐calprotectin, and albumin.

### *Serum drug concentrations and antidrug antibody assay*

Blood was drawn for the determination of serum concentrations of ADL and ADAs regardless of the injection time. The drug was measured using a validated in‐house assay in which biotinylated recombinant TNF‐α is used to trap ADL onto a streptavidin‐coated solid phase. The captured drug was then quantified using an EU^3+^‐labeled protein‐A tracer and time‐resolved fluorescence. The assay for serum ADL concentrations is fully automated on the AutoDELFIA® (PerkinElmer, Waltham, MA, USA) immunoassay platform. ADAs to ADL were analyzed using in‐house inhibition assays that only measure neutralizing ADAs (antibodies that inhibit the TNF‐binding capacity of the drugs).^18^ ADAs were not analyzed in samples with ADL serum concentrations > 5 mg/L as high drug concentrations interfere with the assays for ADAs.

### *Statistical analysis*

The normality of the data was tested using the Shapiro‐Wilk test. Medians and ranges were reported for continuous data as the data were not normally distributed. The Mann–Whitney test was used to test for differences between groups. Numbers and percentages were reported for categorical data, and the chi‐square test or Fisher's exact test were used to test for differences between groups as appropriate. To determine the lower ADL cut‐off concentrations, an ROC analysis was performed separately for CRP, f‐calprotectin, and for composite models (with and without albumin), choosing the point closest to the (0,1) corner (maximizing Youden's index). Based on this, we calculated the sensitivity, specificity, and area under the curve (AUC) with standard error and 95% confidence intervals.

Associations between inflammatory markers and drug levels were explored using multivariable regression analysis with CRP, f‐calprotectin (both log‐transformed to conform to normality), and albumin as dependent variables in three different models and serum adalimumab, age, and sex as independent variables. For all statistical analyses, *p* < 0.05 was considered statistically significant.

All analyses were conducted using SPSS, version 23 (IBM SPSS Statistics for Macintosh, IBM Corp., Armonk, NY, USA) or GraphPad Prism, version 7 (GraphPad Software, La Jolla, CA, USA).

### *Ethics*

The study was approved by the regional ethics committee, REK Vest (2013/554/REK Vest; “IBD i Rogaland—en tverrsnittstudie,” approved April 30, 2013), and was conducted in compliance with the principles expressed in the Declaration of Helsinki. All participating patients provided written informed consent. The study was registered at http://clinicaltrials.gov (NCT02134054).

## Results

### *Patients*

The study included 101 of 108 eligible patients who were on ADL maintenance treatment. The study lost one patient to follow‐up at another center, one patient was considered ineligible for participation due to mental illness, and five patients declined to participate.

The median age was 35 years, and 48 (47%) patients were male. The median time since diagnosis was 9 years, and the median duration of treatment with ADL was 32 months. In this cohort, 16 (15.8%) patients had been on at least one other biological agent before starting their current ADL treatment. Drug concentrations had been measured in roughly one‐fifth of the patients during the last 6 months prior to inclusion, and 13 (12.9%) patients used concomitant immunomodulators (CIMs). Ninety patients received a standardized treatment regimen for ADL (40 mg q2wk), 10 patients 40 mg weekly, and 1 patient 80 mg weekly (Table 1). The f‐calprotectin results were missing for 10 patients, and the serum albumin results were missing for 2 patients.

**Table 1:** Baseline characteristics

Variable	
Age (years)	35 (16–78)
Male, *n* (%)	48 (47%)
Time since diagnosis (years)	9 (0–36)
Duration of treatment (months)	32 (2–112)
SDC (mg/L)	6.9 (0–24.6)
CRP (mg/L)	2.9 (1–45)
f‐Calprotectin (mg/kg)	72 (20–1250)
Albumin (g/L)	39.5 (24.9–47.6)
HBI	2 (0–11)
Disease distribution*, n* (%)	
Upper GI (L4)	1 (1.0%)
Ileal (L1)	42 (41.6%)
Colonic (L2)	19 (18.8%)
Ileocolonic (L3)	39 (38.6%)
Phenotype, *n* (%)	
Nonstricturing, nonpenetrating (B1)	82 (81.2%)
Stricturing (B2)	8 (7.9%)
Penetrating/Perianal disease (B3)	11 (10.9%)
Previous exposure to biologics*, n* (%)	16 (15.8%)
SDC 6 months prior to inclusion, *n* (%)	15 (14.9%)
Detectable ADAs, *n* (%)	7 (6.9%)
Nonstandard dosing (> 40 mg 2qwk) *n* (%)	11 (10.9%)
Medication*, n* (%)	
Corticosteroids	2 (2.0%)
Antibiotics	1 (1.0%)
Immunomodulators	13 (12.9%)
Smoking status, *n* (%)	
Current	19 (18.8%)
Previous	21 (20.8%)

Values are absolute numbers or medians (ranges).

ADA, antidrug antibodies; CRP, C‐reactive protein; F‐calprotectin, fecal calprotectin; HBI, Harvey‐Bradshaw Index; SDC, serum drug concentration.

### *Serum drug concentrations*

For all patients, the median [range] drug concentration was 6.9 mg/L [0–24.6], with no sex difference. There were no differences in drug concentrations between patients who had been on previous biological medication *versus* those who had not (7.6 mg/L *vs* 6.8 mg/L, *P* = 0.63) or between those who had measured drug concentrations before inclusion *versus* those who had not (6.9 mg/L *vs* 6.8 mg/L, *P* = 0.84). Use of CIMs *versus* no use of CIMs did not affect the drug concentrations (6.7 mg/L *vs* 7.0 mg/L, *P* = 0.65).

### *Inflammatory markers*

The median [range] values were as follows: CRP, 2.9 mg/L [1–45]; f‐calprotectin, 72 mg/kg [20–1250]; and albumin, 39.5 g/L [24.9–47.6]. The levels of the inflammatory markers were not significantly influenced by previous exposure to biologics, previous drug concentration measurement, or CIM use.

### *Associations between serum drug levels and inflammatory markers*

Three different multivariable regression models using, respectively, CRP, f‐calprotectin, and albumin as dependent variables and serum ADL levels, age, and sex as independent variables showed modest but significant negative associations between CRP and ADL levels (*r* ^2^ = 0.10, *P* = 0.002), between f‐calprotectin and ADL levels (*r* ^2^ = 0.16, *P* < 0.005), and a positive association between albumin and ADL levels (*r* ^2^ = 0.14, *P* = 0.001) (Table 2).

**Table 2:** Age‐ and gender‐adjusted regression analyses for serum adalimumab

	CRP			Fecal calprotectin			Albumin		
	*β*	*P*‐value	*R* ^*2*^	*β*	*P*‐value	*R* ^*2*^	*β*	*P*‐value	*R* ^*2*^
Adalimumab	−0.06	0.002	0.10	−0.08	<0.0005	0.16	0.27	0.001	0.14
Age	0.002	0.80		−0.01	0.32		−0.04	0.17	
Sex	−0.20	0.50		−0.21	0.35		−1.20	0.13	

CRP, C‐reactive protein.

### *Inflammatory markers and serum drug concentrations*

#### *C‐reactive protein*

**Figure 1:**
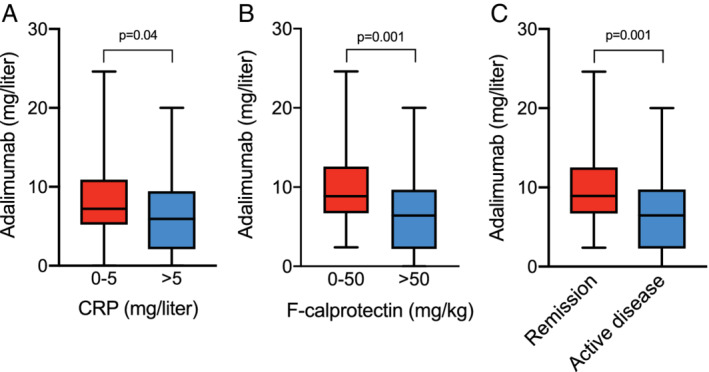
Adalimumab concentrations (mg/L) in patients with Crohn's disease with (a) C‐reactive protein (CRP) 0–5 mg/L (remission, *n* = 71), CRP > 5 mg/L (active disease, *n* = 30). (b) Fecal calprotectin 0–50 mg/kg (remission, *n* = 34), fecal calprotectin > 50 mg/kg (active disease, *n* = 57). (c) CRP 0–5 mg/L and fecal calprotectin 0–50 mg (remission, *n* = 31), CRP > 5 mg/L and/or fecal calprotectin > 50 mg/kg (active disease, *n* = 60). Medians and ranges are shown.

The patients were divided into two groups based on CRP concentrations: 0–5 mg/L (remission, *n* = 71) and > 5 mg/L (active disease, *n* = 30). These subgroups had significantly different median [range] drug concentrations: 7.2 mg/L [0–24.6] *versus* 6.0 mg/L [0–20], *P* = 0.04 (Fig. 1a).

#### *f‐Calprotectin*

As for the CRP analysis, the patients were divided into two subgroups based on f‐calprotectin levels: 0–50 mg/kg (remission, *n* = 34) and > 50 mg/kg (active disease, *n* = 57). These subgroups also had significantly different drug concentrations: 8.9 mg/L [2.4–24.6] *versus* 6.4 mg/L [0–20], *P* = 0.001 (Fig. 1b).

#### *Composite variable of CRP and f‐calprotectin*

For the combined variable of CRP and f‐calprotectin, patients were divided into two subgroups: CRP 0–5 mg/L and f‐calprotectin 0–50 mg/kg (remission, *n* = 31) *versus* CRP > 5 mg/L and/or f‐calprotectin > 50 mg/kg (active disease, *n* = 60). The drug concentrations in these two groups were significantly different, 8.9 mg/L [2.4–24.6] *versus* 6.5 [0–20], *P* = 0.001 (Fig. 1c).

### *ROC analysis*

#### *C‐reactive protein*

**Figure 2:**
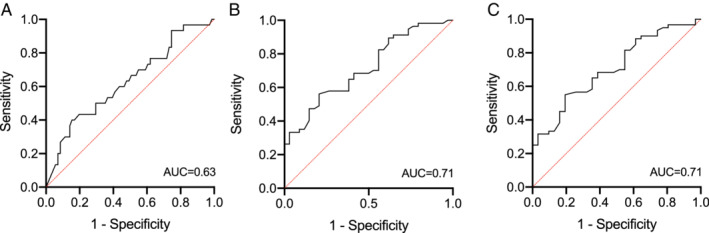
Receiver operating characteristic (ROC) curve analysis of adalimumab concentrations in (a) Patients with C‐reactive protein (CRP) > 5 mg/L representing active disease. (b) Patients with fecal calprotectin > 50 mg/kg representing active disease. (c) Patients with and without CRP > 5 mg/L and/or fecal calprotectin > 50 mg/kg representing active disease. AUC, area under the curve.

Using a cut‐off level of 5 mg/L for CRP with values > 5 mg/L representing active disease, an ROC analysis gave an AUC of 0.63 (std. error 0.062, *P* = 0.04, 95% CI 0.51–0.75). In terms of optimizing sensitivity and specificity, the optimal lower cut‐off value for therapeutic serum concentration was 5.7 mg/L, with a sensitivity of 70% and a specificity of 50% (Fig. 2a).

#### *f‐calprotectin*

We used a cut‐off level of 50 mg/kg for f‐calprotectin, with values > 50 mg/kg representing active disease. An ROC analysis gave an AUC of 0.71 (std. error 0.05, *P* < 0.001, 95% CI 0.61–0.82). In terms of optimizing sensitivity and specificity, the optimal lower cut‐off value for therapeutic serum concentration was 6.9 mg/L, with a sensitivity of 71% and a specificity of 58% (Fig. 2b).

#### *Composite variable of CRP and f‐calprotectin*

An ROC analysis gave an AUC of 0.71 (std. error 0.06, *P* = 0.001, 95% CI 0.60–0.82). In terms of optimizing sensitivity and specificity, the optimal lower cut‐off value for therapeutic serum concentration was 6.8 mg/L, with a sensitivity of 74% and a specificity of 57% (Fig. 2c).

### *Albumin*

#### *ROC analysis of composite variable CRP, f‐calprotectin, and albumin*

Serum albumin quartile levels were as follows: Q1: 24.9–36.4 g/L; Q2: 36.5–39.5 g/L; Q3: 39.6–41.4 g/L; and Q4: 41.5–47.6 g/L.

Two models were developed according to the different selected albumin cut‐off levels.

**Figure 3:**
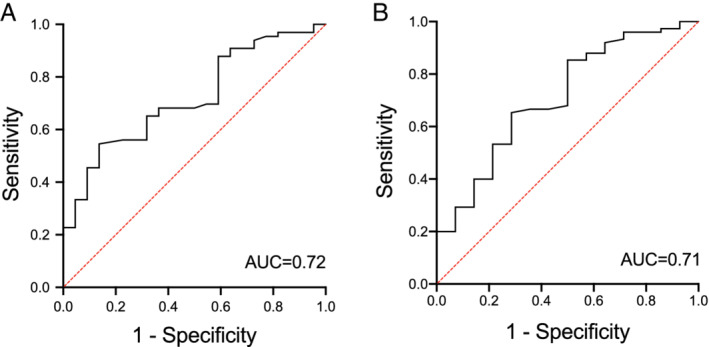
Receiver operating characteristic (ROC) curve analysis of adalimumab concentrations in (a) Patients with C‐reactive protein (CRP) > 5 mg/L and fecal calprotectin > 50 mg/kg and albumin < 36.5 g/L representing active disease. (b) Patients with CRP > 5 mg/L and fecal calprotectin > 50 mg/kg and albumin < 41.5 g/L representing active disease. AUC, area under the curve.

Adding albumin ≥ 36.5 g/L to *CRP* 0–5 mg/L and f‐calprotectin 0–50 mg/kg, an ROC analysis gave an AUC of 0.72 (std. error 0.06, *P* = 0.002, 95% CI 0.61–0.84). In terms of optimizing sensitivity and specificity, the optimal lower cut‐off value for therapeutic serum concentration was 6.8 mg/L, with a sensitivity of 77% and a specificity of 56% (Fig. 3a).

For albumin ≥ 41.5 g/L, a composite ROC analysis gave an AUC of 0.71 (std. error 0.08, *P* = 0.01, 95% CI 0.56–0.86). In terms of optimizing sensitivity and specificity, the optimal lower cut‐off value for therapeutic serum concentration was 7.0 mg/L, with a sensitivity of 71% and a specificity of 53% (Fig. 3b).

## Discussion

In this study, we determined a lower ADL cut‐off concentration of 6.8 mg/L to be associated with biochemical remission in Crohn's disease, using CRP and f‐calprotectin as a composite surrogate disease activity marker. Similarly, using separate inflammatory marker models, we found cut‐off concentrations of 5.7 mg/L and 6.9 mg/L for CRP and f‐calprotectin, respectively. In two exploratory composite models, we also added serum albumin to CRP and f‐calprotectin, showing lower thresholds of 6.8 mg/L (using albumin cut‐off ≥ 36.5 g/L) and 7.0 mg/L (using albumin cut‐off ≥ 41.5 g/L). Thus, we demonstrated separate cut‐off values for ADL concentrations that were statistically significant using all three inflammation markers; dividing patients based on their levels of the selected disease activity marker appeared to be useful for developing ROC models. The significant associations between ADL and investigated disease activity markers supported these findings.

Previous studies have also used conventional biomarkers of inflammation in Crohn's disease to examine ADL concentration thresholds with the aim of identifying the lowest drug concentration required to maintain disease remission. CRP has been widely used as such a biomarker,^19^, ^20^, ^21^, ^22^ and studies have demonstrated an association between CRP normalization and ADL concentrations, finding lower cut‐off levels in the range of 5.6–8.5 mg/L.^23^, ^24^, ^25^, ^26^, ^27^ These results are in line with our findings.

The f‐calprotectin protein is another well‐documented marker of disease activity in Crohn's disease.^28^, ^29^, ^30^, ^31^ One previous study found a lower ADL threshold of 7.2 mg/L using f‐calprotectin levels of 59 mg/kg as indicative of active disease.^27^ Both the f‐calprotectin level used and the lower ADL cut‐off determined are in line with our results.

Only one previous study explored a combined disease activity marker of CRP (< 5 mg/L) and f‐calprotectin (< 250 mg/kg), determining a lower ADL cut‐off of at least 9.5 mg/L. However, the referred study only consisted of 34 patients, and the result did not reach statistical significance (*P* = 0.44).^32^

Serum albumin levels have not been widely used as a marker of disease activity in Crohn's disease. However, an association has been reported,^33^, ^34^, ^35^ and one study showed an association between ADL concentrations and albumin levels (albumin levels < 40 g/L were considered indicative of active disease), with a lower ADL cut‐off level of 7.0 mg/L.^26^ Our experimental composite variable consisting of CRP, f‐calprotectin, and serum albumin yielded concurrent ADL cut‐offs of 6.8 mg/L and 7.0 mg/L. This composite model also produced the highest AUC, although it was only slightly improved compared with the uni‐ and bivariate models. The patients in this study had a considerable disease duration (median of 9 years) and were on long‐term maintenance treatment (median duration of 32 months). Thus, the majority of patients were probably enduring responders with low or no disease activity as reflected by their low median concentrations of CRP and f‐calprotectin. Interestingly, we were still able to define a lower cut‐off concentration of ADL that distinguished between remission and active disease. This indicates that proactive monitoring of ADL concentrations in clinical practice could be meaningful to retain remission in the long‐term follow‐up of patients with Crohn's disease, not only after commencing ADL therapy.

Although it is difficult to make direct comparisons between the ADL thresholds in our study and in previous reports due to differences in the serum drug assays and the methodology, as well as in the ROC curve analysis (AUC) and in the selection of specificity and sensitivity, there are still consistent similarities with our threshold findings in the range of 5.8–7.0 mg/L.

On the other hand, one recent study using a CRP level of 5 mg/L reported a considerably higher ADL threshold of 11.8 mg/L.^36^ This could be explained by more severe disease (i.e. a higher frequency of fistulizing disease, as well as high‐dose treatment) in that patient population.

A strength of our study was the large sample size of 101 patients, making it larger than previous studies. This study also has some limitations, including its cross‐sectional design. We did not perform colonoscopies to investigate endoscopic inflammation, so the true rate of remission, that is, mucosal healing, was therefore unknown. Blood samples to analyze ADL concentrations were drawn randomly during the treatment interval. Although ADL levels appear to be stable at different time points between injections,^27^, ^37^, ^38^ one study suggested that ADL levels are lower at the end of treatment cycles, favoring the use of true trough measurements for ADL.^39^ Finally, although the ROC analyses yielded significant AUCs, this method has some degree of uncertainty, including extrapolation of the ROC curve and the model used for curve fitting.

In conclusion, CRP and f‐calprotectin may be useful markers of clinical disease activity in order to identify a lower ADL threshold. Serum albumin appears to be a promising marker in this setting but needs further validation. A serum ADL concentration of at least 6.8 mg/L appears favorable for maintaining biochemical remission in Crohn's disease, supporting a proactive role of TDM in Crohn's disease patients on ADL treatment.
